# A role of *ygfZ *in the *Escherichia coli *response to plumbagin challenge

**DOI:** 10.1186/1423-0127-17-84

**Published:** 2010-11-09

**Authors:** Ching-Nan Lin, Wan-Jr Syu, Wei-Sheng W Sun, Jenn-Wei Chen, Tai-Hung Chen, Ming-Jaw Don, Shao-Hung Wang

**Affiliations:** 1 Institute of Microbiology and Immunology, National Yang-Ming University, Taipei, 112 Taiwan; 2 National Research Institute of Chinese Medicine, Beitou 112, Taipei, Taiwan; 3 Department of Microbiology, Immunology and Biopharmaceuticals, National Chiayi University, Chiayi 600, Taiwan

## Abstract

Plumbagin is found in many herbal plants and inhibits the growth of various bacteria. *Escherichia coli *strains are relatively resistant to this drug. The mechanism of resistance is not clear. Previous findings showed that plumbagin treatment triggered up-regulation of many genes in *E. coli *including *ahpC, mdaB, nfnB, nfo, sodA, yggX *and *ygfZ*. By analyzing minimal inhibition concentration and inhibition zones of plumbagin in various gene-disruption mutants, *ygfZ *and *sodA *were found critical for the bacteria to resist plumbagin toxicity. We also found that the roles of YgfZ and SodA in detoxifying plumbagin are independent of each other. This is because of the fact that ectopically expressed SodA reduced the superoxide stress but not restore the resistance of bacteria when encountering plumbagin at the absence of *ygfZ*. On the other hand, an ectopically expressed YgfZ was unable to complement and failed to rescue the plumbagin resistance when *sodA *was perturbed. Furthermore, mutagenesis analysis showed that residue Cys228 within YgfZ fingerprint region was critical for the resistance of *E. coli *to plumbagin. By solvent extraction and HPLC analysis to follow the fate of the chemical, it was found that plumbagin vanished apparently from the culture of YgfZ-expressing *E. coli*. A less toxic form, methylated plumbagin, which may represent one of the YgfZ-dependent metabolites, was found in the culture supernatant of the wild type *E. coli *but not in the Δ*ygfZ *mutant. Our results showed that the presence of *ygfZ *is not only critical for the *E coli *resistance to plumbagin but also facilitates the plumbagin degradation.

## Background

5-Hydroxy-2-methyl-1,4-naphthoquinone (5-hydroxyl-2-methyl-naphthalene-1,4-dione, IUPAC), known as plumbagin, is found in many herbal plants. It has been found to have antibacterial [1], antifungal [2], anticancer [3], and antimutagenic activities [4]. Similar to redox-cycling chemicals such as paraquat and menadione (vitamin K3), plumbagin generates superoxide or reactive oxygen species that trigger the oxidative stress response [5]. The genes controlled by *oxyR *and *mar/sox *are known as the major regulons responsive to the oxidative stress in bacteria. In subtle differences, *oxyR *is robustly activated in response to oxidative stress [6] while *mar/sox *are activated by inhibition of the MarR repressor [7] and by oxidization of SoxR [8,9]. Currently, several lines of evidence suggest that the toxicity of plumbagin is not simply due to production of reactive oxygen species. Plumbagin modifies the lactose carrier, which results in a loss of galactoside-binding ability [10]. Furthermore, high concentration of plumbagin (greater than 100 μM) disrupts bacterial respiratory activity through inactivation of NADH dehydrogenase [11].

In a previous proteomic analysis, plumbagin has been shown to up-regulate the expressions of many proteins belonging to the *oxyR *and *mar/sox *regulons in *E. coli*, such as AhpC, MdaB, NfnB, Nfo, SodA, YggX and YgfZ [12]. The function of AhpC, alkyl hydroperoxidase C, is to detoxify endogenous and exogenous peroxides [13]. MdaB (modulator of drug activity B) and NfnB (a predicted oxygen insensitive NAD(P)H nitroreductase) are members of the *mar *regulon [14,15]. The gene *nfo *encodes endonuclease IV, which participates in the repair of H_2_O_2_-induced DNA lesions [16]. SodA, a manganese-containing superoxide dismutase, scavenges and coverts O_2_^- ^to H_2_O_2 _[17]. YggX, an iron-binding protein that is involved in intracellular Fe(II) trafficking, is induced by oxidative stress in order to protect DNA from damage [18,19]. Genes *nfo, sodA, yggX *and *ygfZ *are regulated by marbox sequences that are evidently driven by SoxS [12,20,21]. Genetic deletion of *ygfZ *in *E. coli *has been reported to affect the bacterial tRNA modification and initiation of chromosomal replication [22]. Analysis of the crystallized structure of YgfZ has suggested that the protein may participate in one-carbon metabolism that involves folate or folate derivatives [23]. While *ygfZ *is regulated by SoxS [12], the role of YgfZ in bacteria facing the challenge of plumbagin remains unresolved.

Theoretically, the above types of responses are triggered in order to resolve an immediate threat of the stress. In such circumstances, plumbagin-responsive genes are likely to be involved in either eliminating the toxicity of the chemical or repairing the damage caused by the drug. It is not known whether any of these plumbagin-responsive genes are directly involved in the detoxification of plumbagin. In this study, we identified the genes that are required for *E coli *to resist plumbagin by analyzing the growth of various *E. coli *mutants in the presence of plumbagin. We demonstrated that, among these plumbagin-responsive genes, *ygfZ *and *sodA *are the ones required for counteracting plumbagin toxicity. Furthermore, we provided evidence that YgfZ is needed for the degradation of plumbagin. A methylated and less toxic compound found in the media may represent one of the degradation products. Molecularly, Cys228 in the conserved region of *E. coli *YgfZ is essential for this anti-plumbagin activity.

## Methods

### Bacterial strains, chemicals, and culture conditions

Mutants of *E. coli *K12 with single gene disruption at *ahpC, marA, mdaB, nfnB, nfo, sodA, soxS, soxR, ygfZ, yggX, and lpp*, respectively, were gifted from Dr. Hirotada Mori at Nara Institute of Science (Japan), and the parental strain BW25113 was used as the wild-type strain in all comparison experiments. The genotype of BW25113 is *lacI^q ^rrnB_T14 _ΔlacZ_WJ16 _hsdR514 ΔaraBAD_AH33 _ΔrhaBAD_LD78_*. *E. coli *K-12 JM109 was used as the cloning host. Bacteria were cultured in the Luria-Bertani (LB) broth (Difco) at 37°C with vigorous rotating (150 rpm, Firstek Scientific S306R). Plumbagin (Sigma) was dissolved in dimethyl sulfoxide as a 10 mg/ml stock.

### Primers and expression plasmids

Primers used in this study are listed in Table 1. Plasmid pMH-ygfZ has been described previously [12]. To induce the expression of SodA by IPTG, pQE-sodA was constructed by amplifying the *sodA *fragment from the *E. coli *genomic DNA with primers PsodAF and PsodAR; the amplified fragment was then digested with *Ba*mHI and ligated into pQE60 (Qiagen) previously digested with the same enzyme. Similarly, pQE-ygfZ was constructed by PCR amplification of the *ygfZ *fragment using primers PygfZF and PygfZR (Table 1), which was followed by insertion of the fragment into *Nco*I/*Bgl*II-digested pQE60. In this way, two plasmids were created to express the SodA and YgfZ proteins, respectively, both with hexahistidine (His_x6_) tagged at the C-termini. pQE-Kp_ygfZ, and pQE-Mtb_Rv0811c were generated by a similar strategy, except that the genomic DNAs used for amplification were extracted from *Klebsiella pneumoniae *and *Mycobacterium tuberculosis*, respectively, and the primer pairs separately used were PkpygfZF/PkpygfZR and PRv0811cF/PRv0811cR (Table 1).

**Table 1:** Primers used and their sequences

Name	Sequence (5' to 3')	Used in construction
PygfZF	CCATGGCTTTTACACCTTTTCCTCCCCG	pQE-ygfZ

PygfZR	AGATCTCTCTTCGAGCGAATACGGCAGC	

PsodAF	GGACTTATGAGCTATACCCTGCCATC	pQE-sodA

PsodAR	GGATCCTTTTTTCGCCGCAAAACGTA	

PkpygfZF	CCATGGGTATGGCTTTTACACCTTTTCC	pQE-Kp_ygfZ

PkpygfZR	AGATCTATTTTCTTCCAGCGAATACGGC	

PRv0811cF	CCATGGCCGCAGTCCCTGCCCCAGACCC	pQE-Rv_0811c

PRv0811cR	AGATCTCCGAATACCGCCGCGCAGCCGC	

PygfZK226AF	CAGCTTTAAGGCCGGCTGTTATACCG	pQE-ygfZK226A

PygfZk226AR	CGGTATAACAGCCGGCCTTAAAGCTG	

PygfZG227AF	CTTTAAGAAAGCCTGTTATACCGGAC	pQE-ygfZG227A

PygfZG227AR	GTCCGGTATAACAGGCTTTCTTAAAG	

PygfZC228AF	CTTTAAGAAAGGGGCTTATACCGGACAAG	pQE-ygfZC228A

PygfZC228AR	CTTGTCCGGTATAAGCCCCTTTCTTAAAG	

PygfZC228SF	CTTTAAGAAAGGCTCGTATACCGGAC	pQE-ygfZC228S

PygfZC228SR	GTCCGGTATACGAGCCTTTCTTAAAG	

PygfZC228MF	CTTTAAGAAAGGCATGTATACCGGAC	pQE-ygfZC228M

PygfZC228MR	GTCCGGTATACATGCCTTTCTTAAAG	

PygfZY229AF	TAAGAAAGGCTGTGCTACCGGACAAG	pQE-ygfZY229A

PygfZY229AR	CTTGTCCGGTAGCACAGCCTTTCTTA	

PygfZT230AF	AAGGCTGTTATGCCGGACAAGAGATG	pQE-ygfZT230A

PygfZT230AR	CATCTCTTGTCCGGCATAACAGCCTT	

PygfZG231AF	GCTGTTATACCGCGCAAGAGATGGTG	pQE-ygfZG231A

PygfZG231AR	CACCATCTCTTGCGCGGTATAACAGC	

PygfZQ232AF	CTGTTATACCGGAGCAGAGATGGTGG	pQE-ygfZQ232A

PygfZQ232AR	CCACCATCTCTGCTCCGGTATAACAG	

PygfZE233AF	GTTATACCGGACAGGCCATGGTGGCGCGA	pQE-ygfZE233A

PygfZE233AR	TCGCGCCACCATGGCCTGTCCGGTATAAC	

PygfZΔ226-237F	GGGCGGTATCAGCTTTAAGGCCAAATTCC	pQE-ygfZΔ226-237

PygfZΔ226-237R	GGAATTTGGCCTTAAAGCTGATACCGCCC	

PQEF	GGCGTATCACGAGGCCCTTTTCG	Fragment amplification

PQER	CATTACTGGATCTATCAACAGG	Fragment amplification

### Site-directed mutagenesis and deletion

Mutagenesis was carried out by PCR. Construction of a variant of *E. coli *YgfZ (K226A) with Lys at residue 226 replaced with Ala was given as an example. In brief, *ygfZ *in pQE-ygfZ was first PCR amplified separately with two primer pairs, PQEF/PygfZK226AR and PygfZK226AF/PQER (Table 1). Due to the design of the sequences of PygfZK226AR and PygfZK226AF, the two so-amplified PCR products have overlapping termini where the mutated codon is embedded. After mixing and melting the two PCR products, the overlapping regions were annealed to each other. After this, primers PQEF and PQER were added and PCR amplification was carried out to give a fragment containing the full-length *ygfZ *with the designated K226A mutation. The amplicon was then digested with *Nco*I and *Bgl*II, and ligated into a similarly restricted pQE60 vector to give pQE-ygfZK226A. All the other substitution-mutation plasmids that encode the mutated YgfZ variants were constructed in a similar way by selecting appropriate primer pairs (Table 1).

### Immunoblotting

Total protein lysates were prepared as described previously [12]. Electrophoretically separated proteins blotted on nitrocellulose membrane were analyzed by Western blotting using specific antibodies. Anti-YgfZ antibody was generated by immunizing mice with nickel-column purified His_x6_-YgfZ. Rabbit anti-His_x6 _antibody (Bethyl) was used for detecting His_x6_-tagged proteins. Mouse monoclonal anti-DnaK has been described previously [24]. Horseradish peroxidase-conjugated secondary antibodies (Sigma) were used to detect the primary antibodies bound on the membrane. The antibody-bound blots were finally developed using chemiluminescence reagent (Perkin-Elmer) and the signals were obtained by exposing the membrane to X-ray film (Fuji).

### Inhibition zone analysis

Overnight cultures of the various bacterial strains in LB broth were diluted 100-fold into fresh LB broth and grown with aeration at 37°C for 2 h. The turbidity of the cultured bacteria was adjusted to OD_600 _at 0.4 and the resulting bacteria were spread on Mueller-Hinton (MH) agar (Difco) plates using sterile cotton buds. Filter paper discs (8 mm in diameter) containing various chemicals at appropriate amounts were applied to the top of the agar. The diameters of inhibition zones around the filter discs on the plates were measured after overnight incubation at 37°C.

### Minimal inhibitory concentration (MIC) assay

The method described by the Clinical Laboratory Standards Institute (formerly the National Committee for Clinical Laboratory Standards) was followed. In brief, overnight-cultured bacteria in LB broth were diluted 100-fold into MH broth and grown at 37°C for 2 h. The density of refreshed bacteria was adjusted with MH medium to OD_600 _at 0.05. One ml of the diluted bacterial culture was added to 1 ml of MH broth in a glass tube containing an appropriate concentration of plumbagin and then cultured at 37°C with agitation for 20 h. Bacterial turbidity was measured at 600 nm by spectrophotometry.

### Superoxide detection

A previous method [25] was modified to monitor the changes of superoxide level in *E. coli*. In brief, *E. coli *(*lpp*-deleted) was used for transformation with pQE-sodA or pQE-ygfZ. Then, bacteria at early log phase (OD_600 _= 0.4) were loaded with 10 μg/ml of dihydroethidium for 15 min before addition of superoxide inducing agents. Thereafter, the fluorescence of the cultures was followed by monitoring with a fluorescence spectrometer (TECAN) at excitation wavelength 488 nm and emission wavelength 575 nm.

### Isolation of the organic soluble plumbagin metabolite

Overnight culture of the wild-type *E. coli *strain in LB broth was refreshed with aeration at 37°C for 2 h. After adjusting the turbidity to OD_600 _at 0.5, plumbagin was added to the culture to a final concentration at 25 μg/ml. The bacteria were then further agitated at 37°C for 20 h. After removing the bacteria by centrifugation, the spent media (50 ml) were extracted with chloroform (17.5 ml) three times. The combined chloroform extract was dried over anhydrous Na_2_SO_4 _and vacuum-concentrated. The resulted residue was dissolved in minimal chloroform and subjected to high performance liquid chromatography (HPLC) using E. Merck Lobar RP-C18 column (40-63 μm).

### Identification of the structure of plumbagin metabolite

Infrared spectra were obtained with a Nicolet Avatar 320 FTIR spectrophotometer. UV spectra were measured with a Hitachi U-3310 spectrophotometer. Nuclear magnetic resonance spectra were recorded on a Varian VNMRS-600 spectrometer. The electron impact mass spectra were measured with the direct insertion probe on a Finnigan DSQ II mass spectrometer at 70 eV.

### Statistics

All data were taken from at least three independent experiments. Differences between groups were determined using the two-tail Student *t*-test and were considered statistically significant if *p *was < 0.05.

## Results

### *ygfZ *critical for counteracting plumbagin toxicity

**Figure 1:**
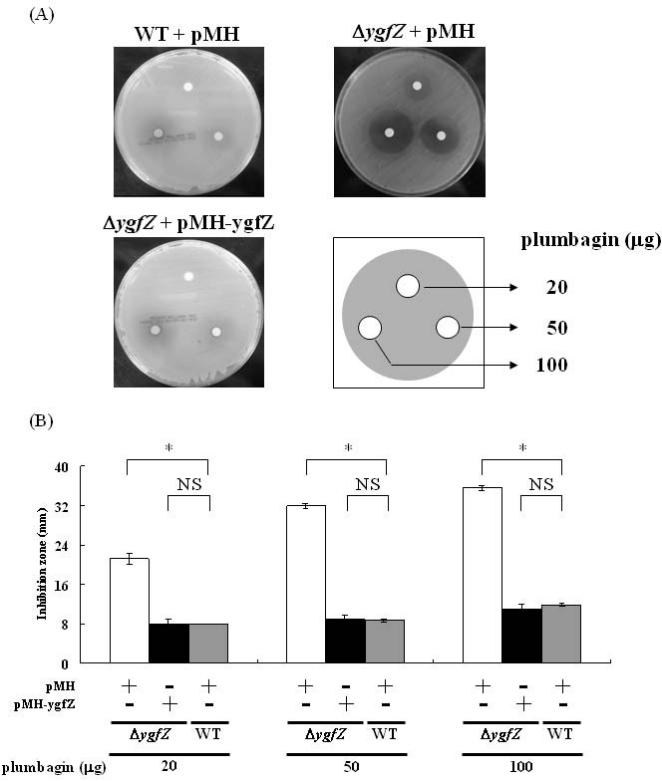
**YgfZ is critical for resolving plumbagin toxicity**. (**A**) Growth inhibition assay on the agar diffusion plates. Bacteria harboring the indicated plasmids were plated overnight at 37°C on MH plates in the presence of plumbagin-containing filter discs (8 mm in diameter). (**B**) Diameters of the inhibition zones seen in (**A**) at different plumbagin concentrations. Note: strain BW25113 (WT) is the parental strain of the Δ*ygfZ *mutant whereas pMH-ygfZ differs from the promoterless pMH vector by carrying *ygfZ *as well its upstream promoter region. NS: no significance; * *p *< 0.05

**Figure 2:**
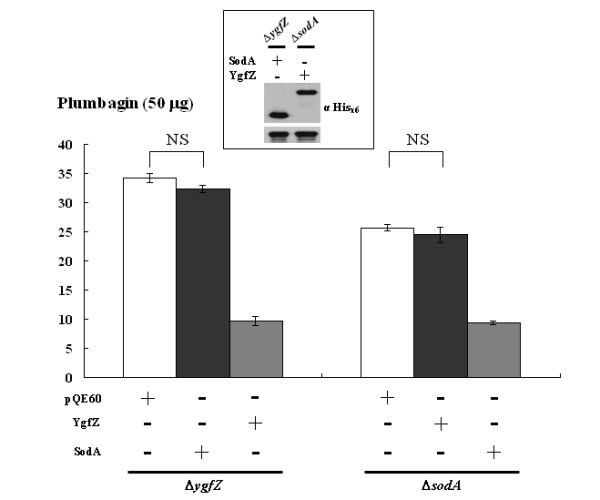
**Different roles played by YgfZ and SodA in counteracting plumbagin**. The Δ*ygfZ *and Δ*sodA *strains were transformed with pQE-sodA and pQE-ygfZ to express SodA and YgfZ, respectively, and the agar diffusion assay was performed similar to that described in legend to Fig. 1. Note: pQE60 was the vector used for expression construction. Inset: the plasmid-encoded His_x6_-tagged proteins were well expressed in the transformants as revealed by Western blotting; antibody-detected DnaK served as a protein-loading control. NS: no significance

To examine the importance of the up-regulated genes previously found [12] in counteracting the plumbagin toxicity, we examined the relative sensitivity of mutant strains with each gene (*ahpC, mdaB, nfnB, nfo, sodA, ygfZ*, and *yggX*) disrupted individually. Also included in these experiments were three strains with similar disruptions at the upstream regulators *soxR, soxS*, and *marA*. The effects on growth inhibition zones surrounding plumbagin-containing discs on the MH agar plates are listed in Table 2. Compared to that of the parental strain, a remarkable increase in plumbagin sensitivity was observed with the Δ*ygfZ *and Δ*sodA *mutants and to a lesser extent with the Δ*soxR*, Δ*soxS*, and Δ*ahpC *strains whereas no effect was seen with the other strains. The MICs of the bacteria toward plumbagin were then determined. The MIC of the parental strain was expectedly much higher than those of the Δ*ygfZ *and Δ*sodA *mutants (Table 3). To ensure that the plumbagin-sensitivity of the Δ*ygfZ *and Δ*sodA *mutants were readily due to the specific gene disruption, complementation assays were carried out. Figure 1A shows a representative result. Upon transformation with pMH-ygfZ, the Δ*ygfZ *mutant showed a diminished inhibition zone, which is similar to that of the parental strain. This reversion of plumbagin-resistance was observed in the presence of different concentrations of plumbagin ranging from 20 to 100 μg per disc (Figure 1B). Similarly, the increased inhibition zone of the Δ*sodA *mutant in an agar diffusion plate could be reduced to that of the wild type by expressing SodA from pQE-sodA (Figure 2, right panel). Therefore, these results confirm that *ygfZ *and *sodA *are involved in the resistance to plumbagin in *E. coli*.

**Table 2:** Growth inhibitory effect of plumbagin against different *E. coli *mutants

Strain tested	Relative sensitivity to plumbagin at different amounts*		
	
	20 μg	50 μg	100 μg
WT, Δ*mdaB*, Δ*nfnB*, Δ*nfo*, Δ*yggX *or Δ*marA*	-	-	-
Δ*soxR*, Δ*soxS, or *Δ*ahpC*	-	-	+
Δ*sodA*	+	++	++
Δ*ygfZ*	+	++	+++

* Bacteria were plated on MH agar plates with plumbagin absorbed on an 8-mm filter paper disc.

-: inhibition zone < 15 mm; +: 15 mm < inhibition zone < 25 mm; ++: 25 mm < inhibition zone < 35 mm; +++: inhibition zone > 35 mm

**Table 3:** MICs for different *E. coli *mutants

Strains	plasmid	MIC (μg/ml)	
		
		plumbagin	methylated plumbagin
WT	-	50	> 200
Δ*sodA*	-	16	> 200
Δ*ygfZ*	-	8	> 200
Δ*ygfZ*/Δ*sodA*	-	4	Not tested
WT	pMH	50	Not tested
Δ*ygfZ*	pMH-ygfZ	50	Not tested
WT	pQE60	40	Not tested
Δ*ygfZ*	pQE-ygfZ	40	Not tested
Δ*ygfZ*	pQE-ygfZK226A	40	Not tested
Δ*ygfZ*	pQE-ygfZG227A	40	Not tested
Δ*ygfZ*	pQE-ygfZC228A	30	Not tested
Δ*ygfZ*	pQE-ygfZC228S	40	Not tested
Δ*ygfZ*	pQE-ygfZC228M	30	Not tested
Δ*ygfZ*	pQE-ygfZY229A	30	Not tested
Δ*ygfZ*	pQE-ygfZT230A	40	Not tested
Δ*ygfZ*	pQE-ygfZG231A	40	Not tested
Δ*ygfZ*	pQE-ygfZQ232A	40	Not tested
Δ*ygfZ*	pQE-ygfZE233A	40	Not tested
Δ*ygfZ*	pQE-ygfZΔ226-237	8	Not tested
Δ*ygfZ*	pQE-Kp_ygfZ	40	Not tested
Δ*ygfZ*	pQE-Rv_0811c	10	Not tested
Δ*ygfZ*	pQE-sodA	8	Not tested
Δ*sodA*	pQE-sodA	40	Not tested
Δ*sodA*	pQE-ygfZ	16	Not tested

### *ygfZ *required for the plumbagin breakdown

To test whether degradation of plumbagin occurs by the bacteria, the amounts of plumbagin remained in the culture media of Δ*ygfZ *and the parental strains were compared by using chloroform extraction and HPLC analysis. After 20-h aerobic cultivation, the concentration of plumbagin remained in the media with the Δ*ygfZ *mutant (5.78 μg/ml) was at least 10 fold higher than that derived from the parental strain (0.49 μg/ml), a fact suggesting a role of *ygfZ *involved in the degradation of plumbagin.

### YgfZ and SodA independently required for resolving plumbagin toxicity

Since both *ygfZ *and *sodA *were found critical for *E. coli *to resolve the plumbagin toxicity, we examined whether they acted independently. Gene *sodA *encodes a manganese superoxide dismutase that converts superoxide anions to molecular oxygen and hydrogen peroxide [26]. As the action of plumbagin has been attributed to superoxide generation [5], SodA is likely to combat plumbagin toxicity by detoxifying the superoxide. On the other hand, in view of the fact that plumbagin is degraded by *E. coli*, it is then reasonable to hypothesize that YgfZ and SodA may counteract plumbagin toxicity in two distinct ways. To test this hypothesis, we addressed whether expressing extra SodA could compensate the absence of YgfZ when *E. coli *is challenged with plumbagin. As shown in Figure 2, when SodA was ectopically expressed from pQE-sodA in the Δ*ygfZ *strain, the inhibition zone remained large and did not differ significantly from that seen with the control plasmid-transformed Δ*ygfZ *strain (Figure 2, left panel). These observations suggest that increasing expression of SodA in bacteria is not sufficient to overcome the plumbagin stress once YgfZ is absent. Reciprocally, increasingly expressed YgfZ in the Δ*sodA *mutant did not reduce the inhibition zone originally seen with the Δ*sodA *strain (Figure 2, right panel). This result indicated that *E. coli*, in the absence of SodA but with ectopically expressed YgfZ, remained incapable of resisting plumbagin toxicity. A doubly mutated strain at both *ygfZ *and *sodA *was then created and MICs toward plumbagin were compared (Table 3). Apparently, the double mutant (Δ*ygfZ/*Δ*sodA*) was the most sensitive strain and its MIC was smaller than either one of the singly disrupted strains. It is then concluded that *ygfZ *and *sodA *both contribute to the resistance of *E. coli *toward plumbagin toxicity but act independently.

**Figure 3:**
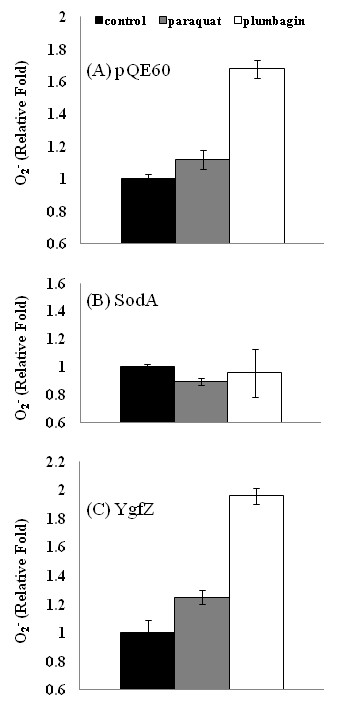
**Superoxide level in *E. coli***. *E. coli *(*lpp*-deleted) was transformed with pQE-sodA and pQE-ygfZ to express recombinant SodA and YgfZ, respectively, and the superoxide levels in bacteria were determined by monitoring the fluorescence changes after loading with dihydroethidium [25]. Data were taken after 120-min treatments with chemicals. (A) Both paraquat (50 μM) and plumbagin (50 μM) stimulated the levels of superoxide detected. (B) The superoxide stimulation seen in (A) was suppressed by SodA expression. (C) The same experiments in (B) were repeated with bacteria expressing YgfZ. Note: pQE60 was the vector control.

To substantiate the notion that different roles are played by YgfZ and SodA in facing the plumbagin challenge, the superoxide levels in the bacteria after receiving chemicals were followed by monitoring the fluorescence change of dihydroethidium. Figure 3A shows that plumbagin tended to increase the superoxide level in bacteria as the known superoxide generator paraquat did. On the other hand, when the bacteria ectopically produced SodA, the original stimulation of superoxide production by either paraquat or plumbagin diminished (compare Figure. 3A with 3B). However, this was not the case when *E. coli *was transformed to produce extra YgfZ (Figure 3C); the trend of increasing superoxide production after paraqaut/plumbagin treatment remained the same (compare Figure 3A and 3C). Therefore, these results consolidated the conception that YgfZ behaves in a mechanism different from that of SodA as to resolving the threat of plumbagin. One of the likely roles of YgfZ involved is possibly to accelerate the breakdown of plumbagin.

### Determining the *ygfZ*-dependent metabolites of plumbagin

**Figure 4:**
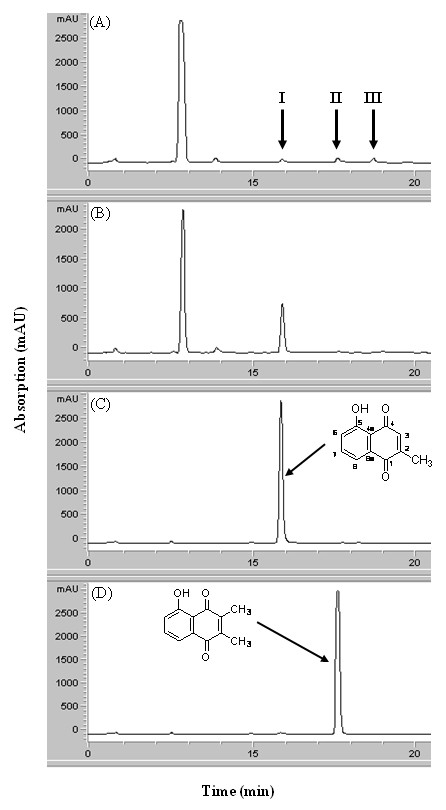
**HPLC analysis of the metabolized plumbagin**. Samples were subjected to RP-C18 column chromatography that was run with a mixture of methanol/H_2_O (7:3, v/v). Compounds eluted were detected with UV absorbance at λ_254_. Samples were chloroform extract of: (**A**) the plumbagin-containing cultivation media of the wild-type *E. coli*; (**B**) the same preparation as (A) but with the Δ*ygfZ *strain; (C) the same preparation as (A) but without bacteria; (D) synthesized 2,3-dimethyl-5-hydroxy-1,4-naphthoquinone extracted from media as described for (C). Compounds identification: I, plumbagin; II, 2,3-dimethyl-5-hydroxy-1,4-naphthoquinone; III, unidentified.

To confirm the plumbagin degradation happened in *E coli*, an effort was made to identify any degraded product of plumbagin. In the HPLC profile of an organic extract prepared from the plumbagin-containing culture media of the parental *E. coli *strain, two extra peaks (peaks II and III in Figure 4A) were found. These peak fractions were collected and subjected to analysis with electron impact mass spectroscopy. A molecule with a molecular weight of 14 Daltons more than that of plumbagin was found from peak II (see Additional file 1-Chemical identification data). Further analysis with nuclear magnetic resonance identified this molecule as 2,3-dimethyl-5-hydroxy-1,4-naphthoquinone (2,3-dimethyl-5-hydroxyl-naphthalene-1,4-dione, IUPAC), whose structure is shown in Figure 4D. This compound is referred as methylated plumbagin hereafter. This compound was then prepared by organic synthesis and compared with that extracted from the spent medium using HPLC (Figure 4A and 4D), infrared, UV and nuclear magnetic resonance analyses. All data obtained supported that the compound from the culture media and that from synthesis were identical. Identification of the compound in peak III was not successful due to a low yield after purification. Furthermore, this methylated plambagin was not seen in the HPLC profile (Figure 4B) generated from the Δ*ygfZ *strain culture and neither found in the repeated experiment.

To examine whether there is any anti-bacterial activity left with methylated plumbagin, MIC was measured, and no apparent activity was found with concentrations up to 200 μg/ml when *E. coli *of the Δ*sodA *and the Δ*ygfZ *strains and the parental strain were tested (Table 3). Therefore, adding a methyl group to the 3-position of naphthoquinone ring apparently diminishes the plumbagin toxicity against *E. coli*.

### Homologues of YgfZ

**Figure 5:**
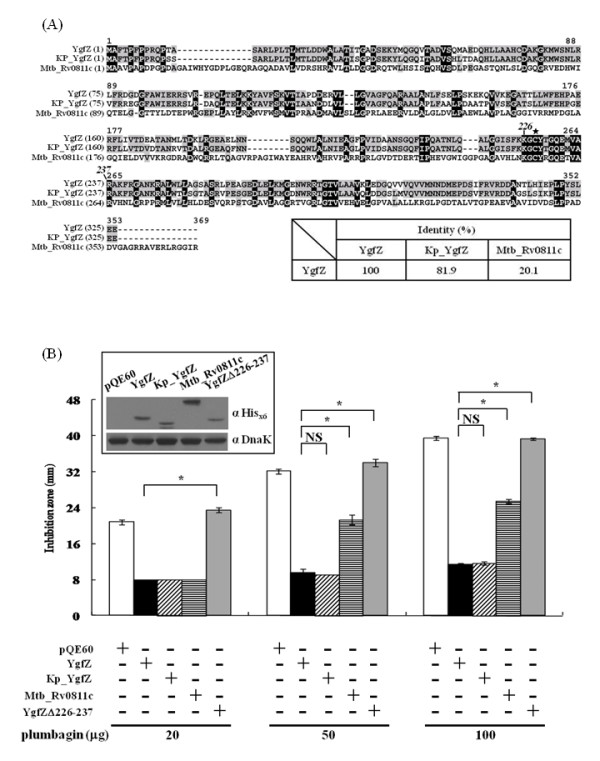
**Complementation to assay the resistance of the Δ*ygfZ *strain toward plumbagin after expressing homologous constructs**. (**A**) Amino-acid-sequence alignment of *E. coli *YgfZ (ref|NP_417374), *K. pneumoniae *YgfZ (Kp_YgfZ; ref|BAH65109), and *M. tuberculosis *Rv0811c (ref|NP_215326). Residues conserved in all three sequences are marked in black whereas those semi-conserved are boxed in gray; labeled above the alignment are residue numbers of the longest Rv0811c sequence and exceptions are those italicized for which represent the YgfZ residues in *E. coli *and *K. pneumoniae*. The cysteine residue in the conserved fingerprint region [23] is asterisked. Inset: amino acid identity between pairs of the three proteins as calculated by Vector NTI (InforMax). (**B**) Comparison of the activities of different YgfZ constructs to support the growth of the Δ*ygfZ E. coli *strain in the presence of plumbagin. Plasmids were separately transformed into the Δ*ygfZ *strain and assayed for the diameters of the growth inhibition zone as in Figure 1B. Inset: the plasmid-encoded proteins expressed in the transformants were detected by Western blotting using anti-His_x6 _antibody; Dank was detected in parallel, to assure a comparable protein loading. Note: pQE60 served as a negative control. NS: no significance; * *p *< 0.05

To analyze the critical region(s) of *ygfZ*, we searched for the conserved residues among the homologues of YgfZ. Alignment of the sequences from *E. coli, K. pneumoniae*, and *M. tuberculosis *is shown in Figure 5A. The identity between the two YgfZ homologues from *E. coli *and *K. pneumoniae *is 81.9%, whereas it is only 20.1% between Rv0811c of *M. tuberculosis *and YgfZ of *E. coli *(insert in Figure 5A). In the agar diffusion assay (Figure 5B), Kp_YgfZ from the *K. pneumoniae ygfZ *was able to restore fully the plumbagin resistance in the *E. coli *Δ*ygfZ *strain. When Mtb_Rv0811c, which is an open reading frame annotated as an aminomethyltransferase-related gene [27], was used in a similar complementation assay, the plumbagin resistance in the Δ*ygfZ *strain was regained partially (Figure 5B). Since there is only a low degree of identity between Rv0811c and YgfZ, it is not clear whether the former is a real counterpart of the latter. Therefore, additional genes annotated as aminomethyltransferases, namely the *gcvT *gene from *E. coli *and *Rv2211c *from *M. tuberculosis*, were cloned and used in similar assays. No function was observed with either of the two constructs. Therefore, it is believed that Rv0811c is the homologue of YgfZ in *M. tuberculosis *and the commonly conserved regions among all sequences must play an essential role.

### Cys 228 in YgfZ critical for plumbagin resistance

Additional experiments were performed to dissect the critical residue(s) in the highly conserved region from K226 to R237, which contains a stretch (^226^K-G-C-Y-T-G-Q-E^233^) of the *E. coli *YgfZ molecule, a region described as fingerprint previously [22,23]. To address the importance of this highly conserved region, amino acid residues 226-237 were deleted and the so-truncated YgfZ was then used in the complementation assay (Figure 5B). The truncated YgfZ totally lost the ability to rescue plumbagin resistance in the Δ*ygfZ *strain. This result is consistent with the expectation that this region is crucial for the YgfZ function.

**Figure 6:**
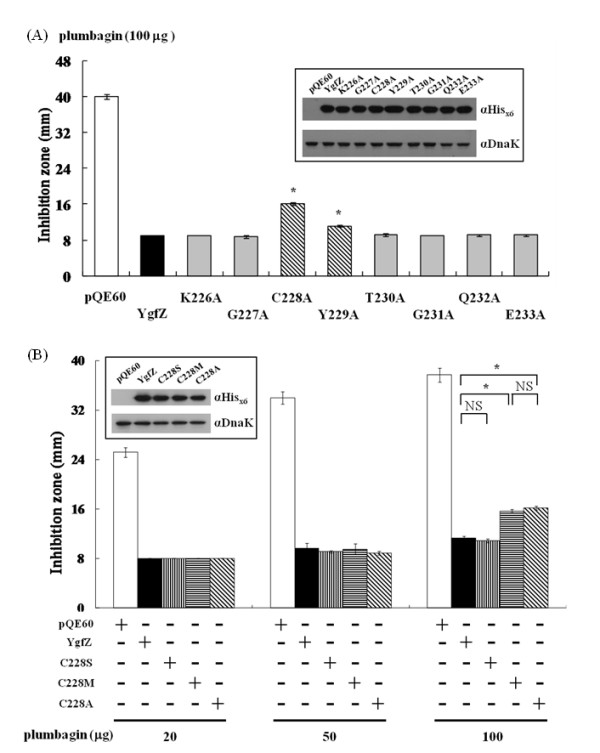
**Analysis of critical residues in the fingerprint region of YgfZ**. (A) Inhibition zone assay for the plumbagin-countering activity of amino acid-substituted YgfZ. The Δ*ygfZ *mutant was transformed with pQE-ygfZ-derived plasmids to express variants of *E. coli *YgfZ. K226A, G227A, C228A, Y229A, T230A, G231A, Q232A, and E233A are constructs with single-amino acid substitution at the indicated residue. Hatched bars mark the substitution mutants with the properties obviously different from the authentic control (black bar). (B) Analysis of the substitutability of C228 with structurally similar amino acids. Complementation transformation of the Δ*ygfZ *mutant was done as in (A) except that plumbagin was applied at three different levels. Note: the construct with the Cys to Ser mutation (C228S) behaved indistinguishable from the authentic YgfZ at all different plumbagin amounts applied while C228 M and C228A mutants apparently deviated from the authentic when plumbagin was applied at 100 μg per disc. Insets: exogenous His_x6_-tagged YgfZ constructs were expressed in the transformed Δ*ygfZ *strain comparably as revealed by Western blotting; DnaK served as a protein-loading control. Note: pQE60 served as negative control. To compare the significance of the data, results from the authentic YgfZ were used as a reference. NS: no significance; * *p *< 0.05

To further narrow down to which residue is critical, single alanine-substitution mutants of YgfZ were created in the fingerprint region. These YgfZ variants were then assessed for the ability to restore plumbagin resistance in the Δ*ygfZ *strain. As shown in Figure 6A, most of these mutated YgfZ constructs (gray bars) readily reduced the inhibition zones and behaved as active as the authentic YgfZ molecule (black bar) in this agar diffusion assay. Two exceptions were mutation at Cys228 and Tyr229 (hatched bars). The C228A mutant performed poorest among these single-point variants. The authentic YgfZ reduced the plumbagin inhibition zone from 40 mm to 10 mm (in diameter), whereas the inhibition zone remained large at 17 mm with C228A and at 12 mm with Y229A (Figure 6A). Not shown in Figure 6A, C228A/Y229A (with double substitutions at residues 228 and 229) lost the complementation activity one step further and resulted in a 28-mm inhibition zone. These results together suggest that C228 is the most critical residue in the fingerprint region of YgfZ followed by Y229 that contributes to the protein's functional integrity but to a lesser extent.

The critical role of C228 in YgfZ was previously predicted to form disulfide bridge [23]. There are two cysteine residues in the *E. coli *YgfZ molecule and the second one is located at residue 63. To test whether C228 is critical for the formation of an intra-molecular disulfide in YgfZ, a single-point mutation at C63 was constructed. The YgfZ variant C63G was found to retain the full authentic YgfZ function in the Δ*ygfZ *complementation assay (data not shown), suggesting that the critical role of C228 in YgfZ does not rely on forming an intra-molecular disulfide bond with C63. Further efforts were made to explore mechanisms of C228 function in YgfZ by replacing C228 with either Ser or Met. The resulting variants C228 S and C228 M were then side-by-side compared with C228A in the Δ*ygfZ *complementation assay. Figure 6B shows that C228 S was able to complement to the same degree as the authentic YgfZ and their plumbagin resistances were indistinguishable at three increasing amounts of plumbagin (from 20 μg up to 100 μg per disc). C228 M, similar to C228A, was indistinguishable from the authentic construct when assayed at 20 μg or 50 μg of plumbagin, but it gave less resistance when plumbagin was applied at 100 μg. Therefore, residues with thiol and hydroxyl groups play equivalent role at position 228 of YgfZ in term of plumbagin resistance and this biological role could only be partially replaced by residues with a methyl group.

## Discussion

Among the *E. coli *genes whose products are up-regulated by plumbagin [12], *ygfZ *and *sodA *readily contribute to resisting the plumbagin's toxicity. When tested with plumbagin at 100 μg per disc, the inhibition zone of the Δ*ygfZ *strain was apparently greater than that of the Δ*sodA *strain (Table 2). On the other hand, when paraquat was applied at 1.28 μg per disc, the Δ*ygfZ *strain showed the same resistance as the parental strain whereas the inhibition zone of the Δ*sodA *strain increased substantially (data not shown). It is known that the expression of *sodA *is elevated when *E. coli *is treated with plumbagin and paraquat separately [12,28]. Up-regulation of *ygfZ *expression also occurs when *E. coli *is treated with plumbagin, but not seen with the paraquat treatment [12,29]. Consistently, we have seen that the superoxide induction resulted from encountering plumbagin were severely repressed by an additional expression of SodA (Figure 3B), but not by YgfZ (Figure 3C). It is then conceivable that in the response to the challenge of plumbagin, *E. coli *could not handle the toxicity simply by increasing the amount of SodA. An additional amour with more YgfZ is apparently needed. The mutual irreplaceable roles of SodA and YgfZ for bacteria to resolve the plumbagin challenge (Figure 2) support the notion that the function of YgfZ is acting independently from SodA.

YgfZ homologues are found among many Gram (-) bacteria and in the mitochondria of eukaryotes but are not found in Archaea [22,23]. No counterpart has been found in Gram (+) bacteria except for those in the chromosomes of high-GC Actinobacteria such as *Streptomyces *spp. and *Mycobacteria *spp. The levels of identity among the YgfZ sequences of the enterobacteria are around 80% or higher whereas that between *E. coli *and *M. tuberculosis *is as low as 20%. Interestingly, the anti-plumbagin activity of these YgfZ homologues seems to be well preserved although to different degrees. A stretch (from K226 to R237 in *E. coli *YgfZ) comprising the previously described fingerprint (K-G-C-Y/F-X-G-Q-E) [23] is conserved across these protein sequences. Within this fingerprint region, we have identified C228 as the most imperative residue for plumbagin detoxification (Figure 6A). However, the effects of single residue site-directed mutants were not as profound as that seen with YgfZΔ226-237, which completely lost its anti-plumbagin ability in Δ*ygfZ *mutant (Figure 5B). Although other possibilities could not be excluded, a worst explanation for these observations is that the structure of YgfZ could be completely distorted when the segment of residues 226-237 was deleted. Nevertheless, when residues at 228 and 229 of YgfZ were simultaneously mutated to Ala in the construct of C228A/Y229A, the effect on YgfZ was further amplified; the inhibition zone was close to 28 mm, a size similar to that seen with the Mtb Rv0811c complementation (Figure 5B). This result revealed that these two residues have synergistic effect for anti-plumbagin activity. The possibility of C228 forming a disulfide linkage [23] has been excluded by the substitution experiment of the second Cys at residue 63, which showed no impact on plumbagin resistance. By substituting the thiol group in C228 with a hydroxyl group, we found authentic YgfZ molecule and the C228 S variant were functionally comparable (Figure 6B). In our mass spectroscopy analysis of a vinyl-palmitic acid-reacted sample, the C228 of *E. coli *YgfZ was found to be labeled with palmitate (data not shown). Therefore, C228 is concluded to possess a free thiol side-chain. Since we have observed that this cysteine residue could be functionally replaced by Ser but only to a partial extent by Met or Ala (Figure 6B), the role of Cys at residue 228 is likely to provide a lone pair of electrons during the spatial molecular interactions.

The resistance of bacteria to antimicrobial agents is mediated by a variety of mechanisms [30]. By protein fractionation, we found that YgfZ is located in the cytoplasmic fraction (see Additional file 2-Localization of the *ygfZ *gene product to the cytoplasm), a fact suggesting that YgfZ is unlikely to be a part of an efflux/influx system. Furthermore, by comparing HPLC profiles of organic extracts prepared from the culture media of the parental bacteria and the Δ*ygfZ *strain, we discovered a possible metabolite of plumbagin, 2,3-dimethyl-5-hydroxy-1,4-naphthoquinone. This methylated plambagin in peak II simply constituted a small portion of the plumbagin metabolites after cultivation for 20 h (compare Figure 4A and Figure 4C), an observation suggesting that there may be more breakdown products not recovered or detected by these processes. The identified 2,3-dimethyl-5-hydroxy-1,4-naphthoquinone appears to be non-toxic to bacteria, up to a concentration of 200 μg/ml (Table 3). In a preliminary experiment, we have found that this compound prepared from our synthesis disappeared gradually when added to the bacterial culture, a fact corroborating the notion that this methylated product is not the final breakdown of plumbagin in *E. coli*.

## Conclusion

We found that YgfZ plays a critical role in plumbagin resistance in *E coli*. Based on our current findings, we suggest that the mechanisms of plumbagin resistance in *E. coli *may involve at least two independent gene products. SodA is induced to resolve the plumbagin-induced oxidation stress whereas YgfZ is induced to facilitate the plumbagin breakdown. The latter mechanism involves at least the methylation of plumbagin that yields non-toxic 2,3-dimethyl-5-hydroxy-1,4-naphthoquinone.

## Competing interests

The authors declare that they have no competing interests.

## Authors' contributions

CNL designed and performed the majority of works in this research. WJS was a research supervisor and coordinator. WWS performed the site-directed mutagenesis assays. JWC generated some expression plamsids and initiated the early works in this research. THC carried out the plumbagin metabolite analysis. CNL and SHW wrote the manuscript. MJD and SHW were research group leaders who contributed to data interpretation. All authors were involved in reviewing and updating the text associated with the manuscript. All authors have read and approved the final manuscript.

## Supplementary Material

Additional file 1 **Chemical identification data**. The general chemical properties, IR and UV absorption spectra and NMR analysis of 2,3-dimethyl-5-hydroxy-1,4-naphthoquinone. Click here for file

Additional file 2 **Localization of the *ygfZ *gene product to the cytoplasm**. Western bolt analysis showed the cytoplasmic distribution of YgfZ in *E. coli*. Click here for file
