# Supplementary material for: A role of ygfZ in the Escherichia coli response to plumbagin challenge
Source: J Biomed Sci. 2010 Nov 9;17(1):84. doi: 10.1186/1423-0127-17-84 (PMC2989944; doi:10.1186/1423-0127-17-84)
Supplement: Additional file 1 — Chemical identification data. The general chemical properties, IR and UV absorption spectra and NMR analysis of 2,3-dimethyl-5-hydroxy-1,4-naphthoquinone. [file 1423-0127-17-84-S1.PDF]

Additional file 1 –Chemical identification data

2,3-Dimethyl-5-hydroxy-1,4-naphthoquinone: orange needles; mp 114-115 °C (EtOAc/hexane); IR (KBr) ν_{max} 3431, 1656, 1636, 1612, 1459, 1363, 1296, 1271, 1205, 1156, 1067, 836, 771, 696 cm^{-1}; UV (MeOH) λ_{max} (log ε) 210 (4.72), 246 (4.21), 273 (4.37), 404 (3.84) nm; ^1H NMR ($CDCl_3$, 600 Mueller-Hinton (MH)z) δ 2.15 (s, 6H, Me x 2), 7.20 (dd, 1H, *J* = 8.4, 1.2 Hz, H-6), 7.54 (t, 1H, *J* = 8.4 Hz, H-7), 7.60 (dd, 1H, *J* = 8.4, 1.2 Hz, H-8), 12.2 (s, 1H, OH); ^{13}C NMR ($CDCl_3$, 150 Mueller-Hinton (MH)z) δ 12.3 (Me), 13.0 (Me), 114.9 (C-4a), 118.9 (C-8), 123.7 (C-6), 132.2 (C-8a), 135.9 (C-7), 143.2 (C-2 or C-3), 144.8 (C-3 or C-2), 161.2 (C-5), 184.2 (C-1), 190.2 (C-4); EI-MS m/z (%) 202 (100) [M^+], 187 (7), 174 (23), 159 (14), 145 (12), 131 (17), 120 (15), 92 (17).
