# Supplementary material for: A role of ygfZ in the Escherichia coli response to plumbagin challenge
Source: J Biomed Sci. 2010 Nov 9;17(1):84. doi: 10.1186/1423-0127-17-84 (PMC2989944; doi:10.1186/1423-0127-17-84)

Additional file 2 –Localization of the *ygfZ* gene product to the cytoplasm

Cultures of bacteria (the Δ*ygfZ* mutant and its parental strain, labeled as WT) were separated into bacterial pellets and spent media. While the media (S) were concentrated, the bacterial pellets were further fractionated into cytoplasm (C), total membrane (tM) and periplasm (P) fractions. Proteins in the individual fractions were resolved with SDS-PAGE followed by Western blot analysis with specific antibodies. DnaK, cytosolic heat shock protein 70; OmpC, outer-membrane porin C; MBP, periplasmic maltose-binding protein. Note: boxed area marks where YgfZ was absent.

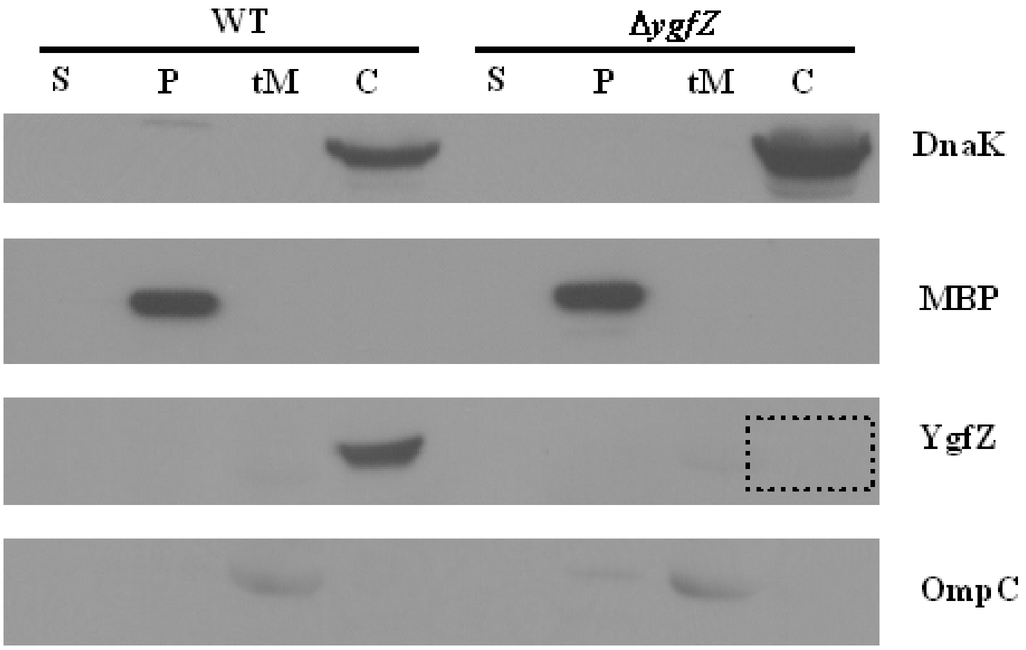

Supplement: Additional file 2 — Localization of the ygfZ gene product to the cytoplasm. Western bolt analysis showed the cytoplasmic distribution of YgfZ in E. coli. [file 1423-0127-17-84-S2.PDF]
